# Heterogeneity among Isolates Reveals that Fitness in Low Oxygen Correlates with *Aspergillus fumigatus* Virulence

**DOI:** 10.1128/mBio.01515-16

**Published:** 2016-09-20

**Authors:** Caitlin H. Kowalski, Sarah R. Beattie, Kevin K. Fuller, Elizabeth A. McGurk, Yi-Wei Tang, Tobias M. Hohl, Joshua J. Obar, Robert A. Cramer

**Affiliations:** a Department of Microbiology and Immunology, Geisel School of Medicine at Dartmouth, Hanover, New Hampshire, USA; b Department of Molecular and Systems Biology, Geisel School of Medicine at Dartmouth, Hanover, New Hampshire, USA; c Department of Chemistry, Keene State College, Keene, New Hampshire, USA; d Department of Medicine, Infectious Disease Service, Memorial Sloan Kettering Cancer Center, New York, New York, USA; e Immunology Program, Sloan Kettering Institute, Memorial Sloan Kettering Cancer Center, New York, New York, USA

## Abstract

Previous work has shown that environmental and clinical isolates of *Aspergillus fumigatus* represent a diverse population that occupies a variety of niches, has extensive genetic diversity, and exhibits virulence heterogeneity in a number of animal models of invasive pulmonary aspergillosis (IPA). However, mechanisms explaining differences in virulence among *A. fumigatus* isolates remain enigmatic. Here, we report a significant difference in virulence of two common lab strains, CEA10 and AF293, in the murine triamcinolone immunosuppression model of IPA, in which we previously identified severe low oxygen microenvironments surrounding fungal lesions. Therefore, we hypothesize that the ability to thrive within these lesions of low oxygen promotes virulence of *A. fumigatus* in this model. To test this hypothesis, we performed *in vitro* fitness and *in vivo* virulence analyses in the triamcinolone murine model of IPA with 14 environmental and clinical isolates of *A. fumigatus*. Among these isolates, we observed a strong correlation between fitness in low oxygen *in vitro* and virulence. In further support of our hypothesis, experimental evolution of AF293, a strain that exhibits reduced fitness in low oxygen and reduced virulence in the triamcinolone model of IPA, results in a strain (EVOL20) that has increased hypoxia fitness and a corresponding increase in virulence. Thus, the ability to thrive in low oxygen correlates with virulence of *A. fumigatus* isolates in the context of steroid-mediated murine immunosuppression.

## IMPORTANCE

*Aspergillus fumigatus* occupies multiple environmental niches, likely contributing to the genotypic and phenotypic heterogeneity among isolates. Despite reports of virulence heterogeneity, pathogenesis studies often utilize a single strain for the identification and characterization of virulence and immunity factors. Here, we describe significant variation between *A. fumigatus* isolates in hypoxia fitness and virulence, highlighting the advantage of including multiple strains in future studies. We also illustrate that hypoxia fitness correlates strongly with increased virulence exclusively in the nonleukopenic murine triamcinolone immunosuppression model of IPA. Through an experimental evolution experiment, we observe that chronic hypoxia exposure results in increased virulence of *A. fumigatus*. We describe here the first observation of a model-specific virulence phenotype correlative with *in vitro* fitness in hypoxia and pave the way for identification of hypoxia-mediated mechanisms of virulence in the fungal pathogen *A. fumigatus.*

## INTRODUCTION

Among both eukaryotic and prokaryotic pathogens, including the pathogenic fungus *Aspergillus fumigatus*, genomic analyses have revealed a startling level of genetic diversity within single species (1–6). An emerging theme in fungal pathogenesis is the extension of this characterization to intraspecies phenotypic heterogeneity. Of particular interest is variation in virulence across clinical and environmental isolates, extending to laboratory strains referenced as “wild-type” (“WT”) strains in pathogenesis studies (7–11). For *A. fumigatus*, studies of phenotypic and virulence heterogeneity have been reported. These include reports of variation in growth *in vitro* (12, 13) and virulence in multiple animal models of aspergillosis, including a *Drosophila melanogaster* model (12), murine models (14), and *Galleria mellonella* larval models (15, 16). Comparisons between two commonly used “WT” laboratory strains, CEA10 and AF293, have begun to reveal differences in the immune response elicited by each strain (17). While this research has started to highlight phenotypic heterogeneity across strains, causal connections between strain genetics, *in vitro* phenotypes, and virulence have yet to be identified. Because virulence is a multifaceted characteristic, both the complex environment of the host and specific factors of the pathogen should be considered to identify mechanisms of pathogenesis and virulence for each host-pathogen interaction (18). Although an ideal antifungal therapeutic would kill the fungus in any host context without extensive collateral damage, mechanistic insights into host context-specific virulence mechanisms are expected to yield novel therapeutic approaches in specific patient populations. To realize this opportunity, the genotypic and phenotypic heterogeneities of both the pathogen and host need to be understood. Consequently, exploring intraspecies genotypic and phenotypic heterogeneity in the *A. fumigatus* populations presents an exciting opportunity to better understand the virulence of this important human pathogen and uncover novel therapeutic strategies in specific patient populations.

The microenvironment of the lung in immunocompromised hosts has been postulated to select for certain *A. fumigatus* genotypes and phenotypes, allowing the fungus to thrive *in vivo*, and has been alluded to in competitive index studies and in the development of drug resistance in clinical strains (19–22). However, the specific selective pressures of various *ex vivo* environments that enable environmental isolates to better respond to the host and increase virulence remain enigmatic (5, 19, 23). Several aspects of the host microenvironment that represent potential selective pressures have been characterized. These include alternations in pH (24, 25), oxygen tensions (26, 27), and nutrient availability (28, 29), among others. While great strides are being made toward understanding how *A. fumigatus* responds to such stresses, utilizing multiple strains to elucidate mechanisms could significantly enhance these efforts.

While assessing the mechanism of virulence across multiple strains *in vivo*, it should be considered that the host microenvironment varies between susceptible populations of patients depending on the state and mechanism of immune suppression. This creates an even more complex interplay of host-pathogen interactions and complicates the understanding of *A. fumigatus* responses to the host (30). While the majority of invasive pulmonary aspergillosis (IPA) cases have been reported among neutropenic individuals, it has recently been recognized that the number of nonneutropenic patients diagnosed with IPA is increasing (31, 32). This shift in IPA patient populations highlights the relevance of the murine triamcinolone model of infection, which presents a different *in vivo* microenvironment than the leukopenic (neutropenic) model (26, 30). For example, we have described variability in tissue oxygen levels between these models, where pulmonary lesions in both models reach oxygen tensions below 1%; however, low oxygen (or hypoxic) lesions in triamcinolone-treated mice, with increased host cell infiltrates, encompass larger areas of tissue than those in leukopenic mice (26).

The ability of *A. fumigatus* to adapt to low oxygen (or hypoxia) is essential for virulence, as the master transcriptional regulator of the hypoxia response in *A. fumigatus*, SrbA, is essential for growth in hypoxia (<5% O_2_) *in vitro* and for virulence in both the triamcinolone and leukopenic murine models of IPA (33, 34). Considering the hypoxic nature of the infected lung, these data suggest that the ability to adapt to low-oxygen environments is critical for growth within the host and virulence. Therefore, we tested the hypothesis that among a heterogeneous group of strains those more fit in low-oxygen environments are more virulent.

Here, we report a strong correlation between *in vitro* hypoxic growth phenotypes and *A. fumigatus* virulence. We observe that the commonly utilized laboratory strains AF293 and CEA10 have markedly different growth phenotypes in liquid culture under normal-oxygen (normoxic) and low-oxygen (hypoxic) conditions. Strikingly, these two strains have a significant difference in virulence in a triamcinolone murine model of IPA despite similar virulence levels within a leukopenic (neutropenic) model. Therefore, we expanded our characterization of *A. fumigatus* strains using a selection of environmental and clinical isolates to test whether the ability to thrive under low oxygen conditions impacts pathogenesis and virulence in the triamcinolone model of IPA. Our characterization of these strains showed heterogeneity across isolates in several *in vitro* growth phenotypes and a correlation between higher hypoxia fitness and increased virulence in this model. In further support of these observations, virulence of a strain with low hypoxia fitness increases with experimental evolution through *in vitro* low-oxygen conditions, supporting a hypothesis that hypoxia fitness is a major factor in *A. fumigatus* virulence in specific host contexts.

## RESULTS

### Two common laboratory strains display virulence heterogeneity in a triamcinolone immunosuppression murine model and varied fitness phenotypes *in vitro*.

**FIG 1:**
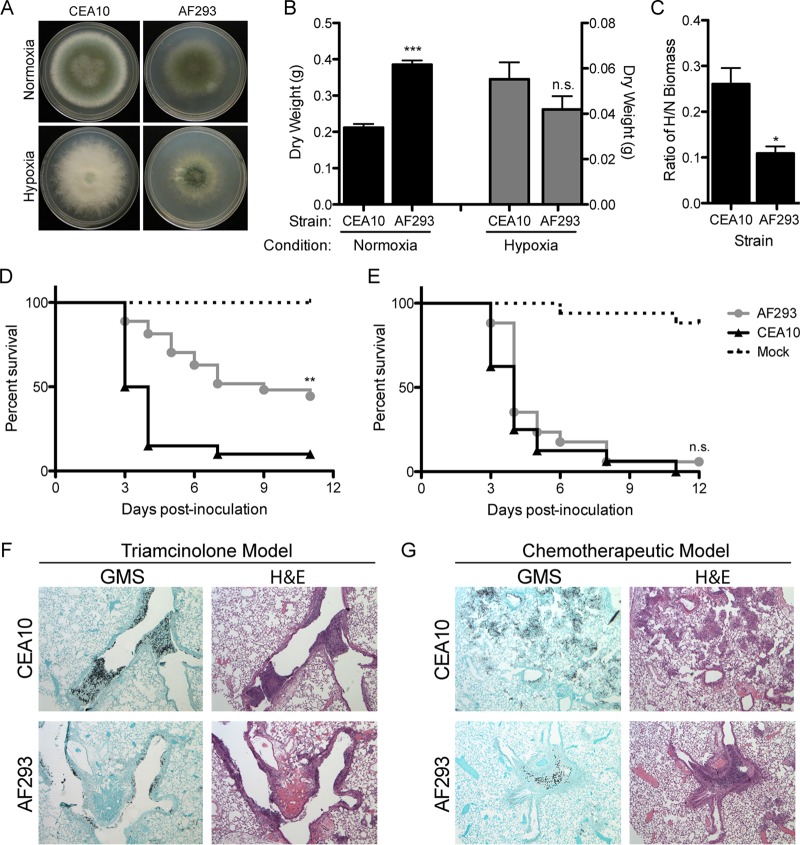
Two common lab strains, CEA10 and AF293, show phenotypic variation and strikingly different virulence in a triamcinolone model of IPA but not a leukopenic model of IPA. (A) Radial growth of CEA10 and AF293 on GMM in normoxia (~21% O_2_) and hypoxia (0.2% O_2_) at 96 h. (B) Biomass from liquid cultures grown in 1% glucose minimal medium (GMM) in normoxia and hypoxia at 48 h. ***, *P* = 0.0004; n.s., not significant by unpaired, two-tailed *t* test. (C) Ratio of biomass in hypoxia to biomass in normoxia, as calculated with values from panel B. *, *P* = 0.0165 by unpaired, two-tailed *t* test. (D) Survival analysis of CEA10 (*n* = 20 from 2 independent experiments) and AF293 (*n* = 27 from 3 independent experiments) in a triamcinolone model of IPA in CD-1 mice inoculated with 2 × 10^6^ conidia intranasally. **, *P* = 0.0002 by log rank test. (E) Survival analysis of CEA10 (*n* = 16) and AF293 (*n* = 17) in a chemotherapeutic model of IPA in CD-1 mice inoculated with 2 × 10^6^ conidia intranasally. n.s., not significant by log rank test. (F and G) Hematoxylin and eosin (H&E) or Gomori methenamine silver (GMS) stains of lungs 3 days postinoculation from triamcinolone-treated mice (F) and chemotherapy-treated leukopenic mice (G) inoculated with 2 × 10^6^ AF293 or CEA10 conidia. Images are representative of three mice, and all error bars indicate standard errors of the means.

Heterogeneity between two commonly used laboratory strains, AF293 and CEA10, has been noted previously by our lab for qualitative hypoxia fitness *in vitro* (35), but the importance of this observation has not been thoroughly explored. Given our previous data highlighting the role of hypoxia fitness in *A. fumigatus* virulence, we sought to quantitatively measure *in vitro* fitness under various oxygen environments for the two “WT” strains. To examine this, we measured radial growth (Fig. 1A) and liquid biomass (Fig. 1B) of CEA10 and AF293 in 1% glucose minimal medium (GMM) in normoxia (ambient air, ~21% O_2_) and hypoxia (0.2% O_2_). On solid medium in hypoxia, CEA10 has significantly greater radial growth than AF293 (*P* < 0.0001) (Fig. 1A). In liquid culture, we found that biomass of AF293 is significantly increased (*P* = 0.0004) in normoxia compared to CEA10 after 48 h. Conversely, CEA10 produces more biomass after 48 h in hypoxia than AF293 (Fig. 1B). To account for the ability of each individual strain to grow in hypoxia relative to normoxia, we calculated the ratio of biomass in hypoxia to biomass in normoxia (H/N) for each strain and observed that the H/N fitness ratio of CEA10 is significantly (*P* = 0.0165) higher than AF293 (Fig. 1C). We conclude that CEA10 has greater hypoxia fitness than AF293.

As we have previously observed extensive low oxygen regions within pulmonary fungal lesions in the murine triamcinolone model of IPA compared to the leukopenic model (26), we hypothesized that the increased *in vitro* hypoxia fitness of CEA10 would allow for increased virulence in the murine triamcinolone model of IPA. In a controlled side-by-side comparison, CEA10 is significantly (*P* = 0.0002) more virulent than AF293 in this model of IPA as quantified by murine mortality (Fig. 1D). However, consistent with previous studies from several laboratories, in the chemotherapeutic model (33), the two strains display nearly identical virulence levels (*P* = 0.2426) as measured by murine mortality, where 90 to 100% of mice succumb to infection by 12 days postinoculation (Fig. 1E). To evaluate the pathology of disease for each strain, we prepared lungs for histological analysis from both models 3 days postinoculation (dpi). Gomori methenamine silver (GMS) staining reveals greater fungal load and denser fungal growth with CEA10 than with AF293 in the triamcinolone and chemotherapeutic models of IPA (Fig. 1F and G). It is also evident that CEA10 demonstrates increased lung parenchyma invasion, whereas AF293 is more often retained in larger airways. Staining with hematoxylin and eosin (H&E) illustrates increased inflammation for both strains in the triamcinolone model compared to the chemotherapeutic model (Fig. 1F and G). Taken together, these data highlight differences in *in vivo* fungal fitness and the inflammatory response between these two laboratory “WT” strains. Based on the observation that the significant increase in the H/N growth ratio of CEA10 corresponds to its increased virulence in the triamcinolone model of IPA, we hypothesized that H/N fitness ratio could be used as a predictor of virulence across *A. fumigatus* strains in the context of steroid-mediated immune suppression.

### *In vitro* hypoxia fitness heterogeneity exists among clinical and environmental isolates.

**FIG 2:**
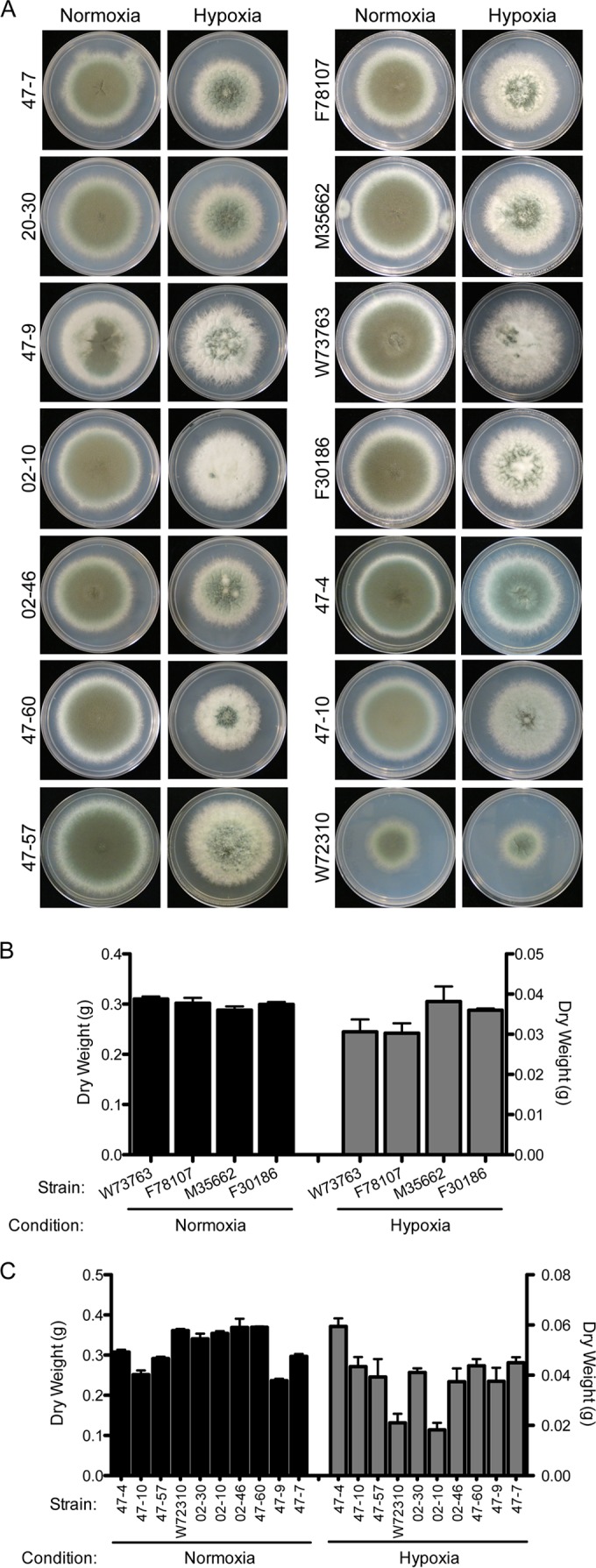
Clinical and environmental isolates show heterogeneity in hypoxic growth. (A) Radial growth of clinical and environmental isolates on GMM under normoxia and hypoxia conditions. (B and C) Four clinical isolates, M35662, F78107, F30186, and W73763 (*n* = 4) (B), and the remaining environmental isolates (*n* = 10) (C) reveal heterogeneity in biomass from liquid culture in normoxia (~21% O_2_) and hypoxia (0.2% O_2_). Data are presented as the means from biological triplicates with error bars representing standard errors of the means.

To test our hypothesis that increased hypoxia fitness might predict the virulence of *A. fumigatus* strains in an immunosuppression-dependent context, we expanded our analysis to a random selection of *A. fumigatus* environmental and clinical isolates confirmed through morphological analysis and sequencing of the calmodulin gene and internal transcribed spacer (ITS) region (data not shown). Four clinical isolates were collected through lung biopsies (M35662 and F78107) and bronchial washes (F30186 and W73763), and 10 environmental isolates were selected from broad geographic regions, including air samples (47-57, 47-60, and 47-9), an indoor hospital sample (47-4), sawmill samples (47-10 and 47-7), and three samples from Netherlands (02-46, 02-10, and 02-30) (Table 1). The 10th environmental isolate is from a sputum sample (W72310) and is included among the environmental isolates, as events of single isolation from sputum are often categorized as environmental contaminants during the collection process (36, 37). As observed with the two “WT” strains, there is a range of fitness phenotypes among the clinical and environmental isolates on solid medium and liquid biomass cultures in both normoxia and hypoxia (Fig. 2A, B, and C). Interestingly, the environmental isolates, in general, show greater variation in biomass under both conditions than the clinical isolates (Fig. 2C). Taken together, these data illustrate that significant hypoxia fitness heterogeneity exists across *A. fumigatus* strains.

**TABLE 1:** Geographic origins of *A. fumigatus* isolates used in this study

Strain	Origin	Source
Lab “wild-type” strains		
AF293	Lung biopsy specimen of neutropenic IPA patient	David Denning Laboratory
CEA10 (CBS144.89)	Patient with IPA	CBS-KNAW Fungal Biodiversity Centre
Environmental isolates		
47-4 (AF250, FA210)	Salford Hope Hospital, Manchester, UK	Paul Dyer, Nottingham, UK
47-10 (AF221)	Sawmill wood, New Zealand	Paul Dyer, Nottingham, UK
47–57 (AFIR957)	Air sample, Dublin, Ireland	Paul Dyer, Nottingham, UK
W72310	Sputum	Memorial Sloan Kettering, NY, USA
47-7 (AF217)	Sawmill, Sweden	Paul Dyer, Nottingham, UK
47-9 (AF70)	Air sample, NJ, USA	Paul Dyer, Nottingham, UK
47-60 (AFRB3)	Air samples, Dublin, Ireland	Paul Dyer, Nottingham, UK
08-19-02-10 (02-10)	Netherlands, Nijmegen	Darius Armstrong, Imperial College London, London, UK^a^
08-19-02-30 (02-30)	Netherlands, Berghem	Darius Armstrong, Imperial College London, London, UK^a^
8-19-02-46 (02-46)	Netherlands, Nijmegen	Darius Armstrong, Imperial College London, London, UK^a^
Clinical isolates		
M35662	Lung biopsy specimen^b^	Memorial Sloan Kettering, NY, USA
F78107	Lung biopsy specimen^b^	Memorial Sloan Kettering, NY, USA
F30186	Bronchial wash	Memorial Sloan Kettering, NY, USA
W73763	Bronchial wash	Memorial Sloan Kettering, NY, USA

a See reference 75 for information on this Netherlands strain.

b These are proven invasive aspergillosis based on modified EORTC criteria.

**FIG 3:**
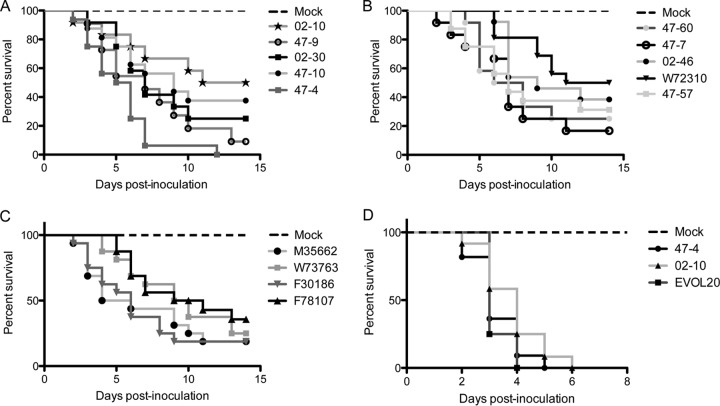
Clinical and environmental isolates present a spectrum of virulence in the triamcinolone model of IPA but not in a chemotherapeutic model. (A and B) Data are separated into two distinct graphs for easy viewing. Triamcinolone-treated CD-1 mice inoculated with 2 × 10^6^ conidia of 02-10 (*n* = 12), 47-9 (*n* = 11), 02-30 (*n* = 12), 47-10 (*n* = 16), 47-4 (*n* = 16), 47-60 (*n* = 12), 47-7 (*n* = 12), 02-46 (*n* = 13), W72310 (*n* = 16), or 47-57 (*n* = 16) reveal a range of virulence levels across the isolates. (C) Clinical isolates (*n* = 16 per strain) inoculated in triamcinolone-treated CD-1 mice at 2 × 10^6^ conidia reveal reduced heterogeneity in virulence (curves not significantly different by log rank test; *P* = 0.2296). (D) Survival of mice treated with both cyclophosphamide and triamcinolone (chemotherapeutic model) were inoculated with 2 × 10^6^ conidia of 47-4, 02-10, or EVOL20 (*n* = 12 per strain). In this model, all three strains produce curves that are indistinguishable from one another (log rank, *P* = 0.1851 for 47-4 and 02-10).

To determine if strain heterogeneity extends to differences in virulence, we assessed virulence of the respective environmental and clinical isolates in the triamcinolone murine model of IPA. Similarly to the *in vitro* phenotypic heterogeneity, the environmental isolates produce a spectrum of virulence, with median survival ranging from 12.5 (W72310) to 5.5 (47-4) dpi (Fig. 3A and B). Among the clinical isolates, the median survival ranges from 5 (M35662) to 10 (F78107) dpi (Fig. 3C). Given that the difference in virulence between CEA10 and AF293 is specific to the triamcinolone model, we assessed virulence of an isolate with hypovirulence in the triamcinolone model, 02-10, and an isolate with hypervirulence in the triamcinolone model, 47-4, in the chemotherapeutic model. Similarly to the “WT” strains, we did not observe a significant difference in virulence between the two isolates in this leukopenic model (*P* = 0.1851 [Fig. 3D]). Taken together, these data illustrate heterogeneity across *A. fumigatus* isolates in *in vitro* growth phenotypes and virulence in a host-specific manner. We hypothesized that like the “WT” strains, the hypoxia fitness of these isolates correlates with the virulence in the triamcinolone model of IPA.

### A higher ratio of hypoxic to normoxic fitness correlates with increased virulence in triamcinolone-treated mice. 

**FIG 4:**
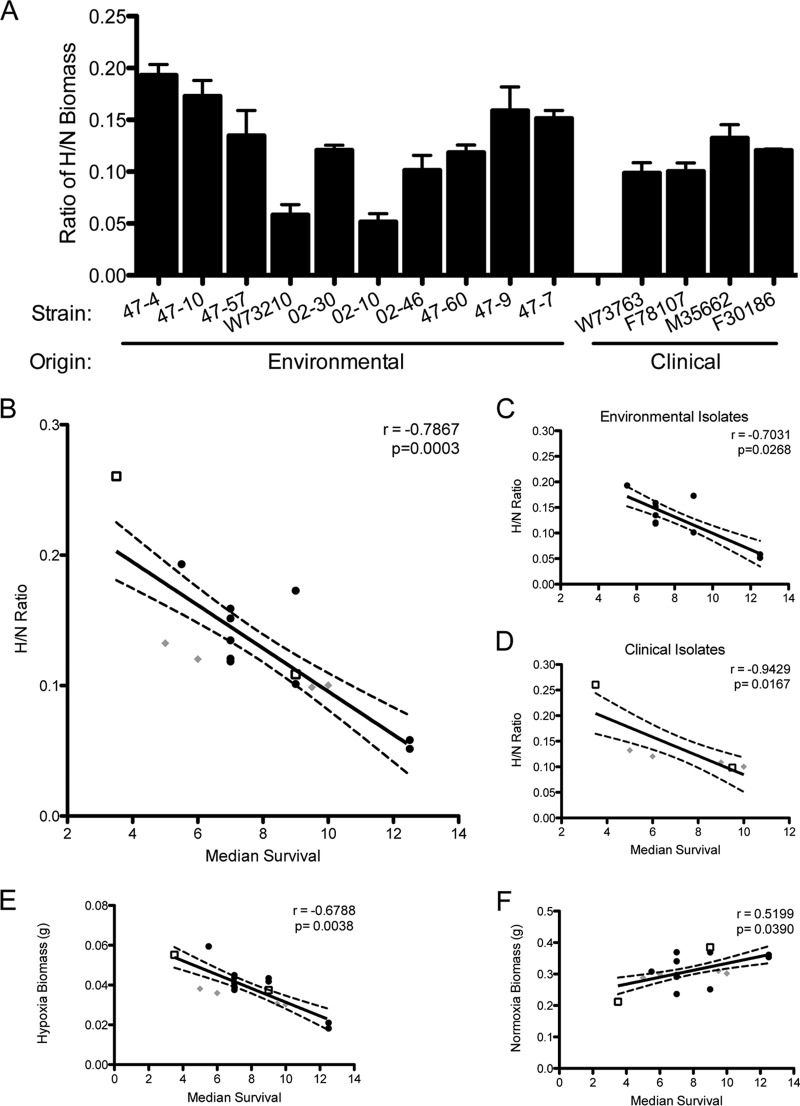
Higher ratio of hypoxia-to-normoxia biomass correlates with increased virulence in the triamcinolone model of IPA. (A) Ratio of liquid culture biomass in hypoxia (0.2% O_2_) to normoxia (~21% O_2_) for environmental and clinical isolates. (B to D) Spearman rank order correlations calculated from H/N ratio and median survival (*n* = 16; *r* = −0.7867; *P* = 0.0003) for all strains (B) (clinical isolates [diamonds], lab-utilized “WT” strains [squares], and environmental isolates [circles]) and separated by environmental (*n* = 10; *r* = −0.7031; *P* = 0.0268) (C) and clinical (*n* = 6; *r* = −0.9429; *P* = 0.0390) (D) isolates. (E and F) Spearman rank order correlations and median survival for all 16 strains of hypoxia-only biomass (*r* = −0.6788; *P* = 0.0038) (E) and normoxia-only biomass (*r* = 0.5199; *P* = 0.0390) (F).

To test whether there is a correlation between hypoxia fitness and virulence in the triamcinolone model of IPA, we determined the H/N ratio of each isolate (Fig. 4A). Among the environmental isolates, the H/N ratio varied from higher, CEA10-like ratios (47-4 and 47-10) to lower AF293-like ratios (W72310 and 02-10). The clinical isolates showed modest variation but interestingly were more similar to each other than the environmental isolates and fell in the middle range of the H/N ratios for CEA10 and AF293 (Fig. 4A). Spearman’s rank order correlation analysis, for nonparametric data (38), of the H/N fitness ratio versus the median murine survival for each strain revealed a significant correlation (Spearman’s rho, *r* = −0.7867, *P* = 0.0003) between the two metrics (Fig. 4B), supporting the hypothesis that increased fitness in hypoxia yields higher virulence in the triamcinolone model of IPA. If we separate isolates into distinct environmental and clinical groups and test the correlation of each group, both remain significant, with a Spearman’s rho (*r*) of −0.7031 (*P* = 0.0268) for the environmental isolates (Fig. 4C) and a Spearman’s rho (*r*) of −0.9429 (*P* = 0.0167 [Fig. 4D]) for the clinical isolates. As controls for nonnormalized growth, we included the Spearman correlation between hypoxic-only and normoxic-only biomass and median survival. The correlations with hypoxia biomass (*r* = −0.6788; *P* = 0.0038 [Fig. 4E]) and normoxia biomass (*r* = 0.5199; *P* = 0.0390 [Fig. 4F]) are both significant but are less robust than the H/N correlation. However, it is important to note that the correlation of normoxic biomass and median survival is positive, indicating that increased growth capacity under normal lab conditions negatively correlates with virulence in this cohort of strains and murine model. Notably, this correlation is host specific, as there is no correlation between median survival in the chemotherapeutic model and the H/N ratio (*r* = −0.2582; *P* = 0.75) with 02-10, 47-4, CEA10, and AF293 (data not shown). Taken together, these data support a hypothesis that fitness in hypoxia, not overall growth in normoxia, correlates with higher virulence of *A. fumigatus* in the triamcinolone model of IPA and that this correlation is host specific.

Interestingly, the environmental isolate 47-10 appears to be an outlier within this correlation, displaying an increased median survival compared to that predicted by the hypoxia fitness ratio. Although correlations reveal connections between two phenotypes, they do not necessarily portray causal relationships. As virulence is a multifaceted characteristic, the outlier 47-10 may utilize alternative mechanisms that account for the high hypoxia fitness without displaying the expected increase in virulence in the triamcinolone model of IPA. Such strains, although outliers within the correlation, can be informative in the identification of traits influencing *A. fumigatus* virulence. One potential mechanism known to influence virulence and host immune responses is cell wall polysaccharide microbe-associated molecular patterns (MAMPs). Moreover, hypoxia is known to dramatically alter the cell wall of *A. fumigatus* and *Candida albicans* (39, 40). However, cell wall integrity analysis of these strains on the cell wall-perturbing agents Congo red (CR) and calcofluor white (CFW) in normoxia and hypoxia does not reveal any obvious phenotypes correlative to virulence (see Fig. S1 in the supplemental material). For example, 47-10 displayed increased resistance to Congo red and CFW in normoxia and hypoxia, similarly to hypoxia-fit and virulent strains 47-4 and CEA10. Ongoing work is focused on uncovering the possible mechanisms of hypoxia-mediated virulence observed in the strains analyzed.

### *In vitro* experimental evolution of a strain with low fitness in hypoxia leads to increased virulence. 

**FIG 5:**
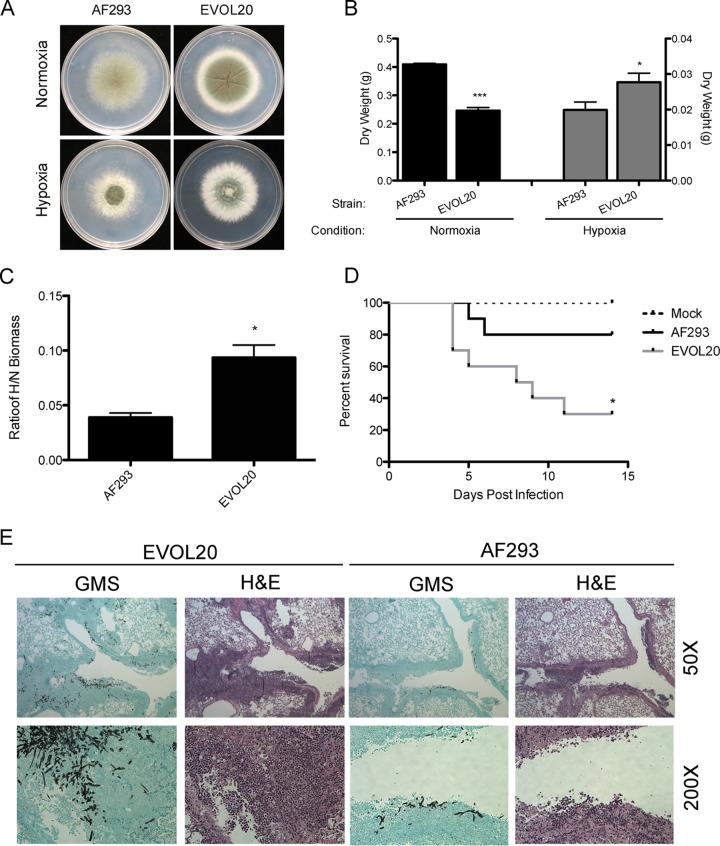
Serial passaging of a strain with low hypoxia fitness increases growth and virulence in triamcinolone-treated mice. (A and B) Radial growth (A) and liquid biomass (B) on GMM in normoxia (~21% O_2_) and hypoxia (0.2% O_2_). ***, *P* = 0.0001; *, *P* = 0.0448, by unpaired, two-tailed *t* test. (C) Ratio of hypoxia biomass to normoxia biomass for EVOL20 compared to the parental AF293 strain. *, *P* = 0.0111 by unpaired, two-tailed *t* test. (D) Survival analysis with a low-dose inoculum (1 × 10^5^ conidia) in the triamcinolone-treated CD-1 mice (*, *P* = 0.0296 by log rank test; *n* = 10 per strain). (E) Hematoxylin and eosin (H&E) or Gomori methenamine silver (GMS) stains of lungs 3 days postinoculation from triamcinolone-treated CD-1 mice inoculated with 1 × 10^5^ conidia. Images are representative of 3 mice.

With the observed correlation between virulence and fitness in hypoxia, we sought to experimentally test the hypothesis that increased fitness in hypoxia promotes virulence. Consequently, experimental evolution was performed by serially passaging the laboratory “WT” strain AF293, which exhibits low fitness in hypoxia (Fig. 1B) and attenuated virulence in the triamcinolone model of IPA (Fig. 1D). Following 20 passages in hypoxia, the resulting *A. fumigatus* strain, named EVOL20, exhibits increased fitness in hypoxia as determined by colony diameter (*P* = 0.0026 [Fig. 5A]) and liquid biomass (*P* = 0.0448 [Fig. 5B]) compared to the parental strain. Interestingly, in normoxia, EVOL20 also displays increased colony density and radial growth on solid medium at 96 h (Fig. 5A) but displays significantly reduced biomass in normoxic liquid cultures compared to AF293 (*P* = 0.0001 [Fig. 5B]). This increased fitness results in a significantly (*P* = 0.0111) increased hypoxia fitness ratio (H/N) of EVOL20 compared to AF293 (Fig. 5C). To determine whether this ratio correlates with increased virulence of the strain, we used a low-dose (1 × 10^5^ conidia) triamcinolone model of IPA and observed that EVOL20 has a remarkably significant increase in virulence over AF293 (*P* = 0.0296 [Fig. 5D]). Histology results from 3 dpi postinoculation reveal greater invasion of EVOL20 into the lung parenchyma and a general increase in fungal growth and inflammation compared to lungs inoculated with AF293 (Fig. 5E). Notably, the EVOL20 strain also displays increased virulence compared to AF293 in the chemotherapeutic model of IPA (*P* = 0.0003); however, the median survival differs only from day 4 for AF293 to day 3 for EVOL20 (Fig. 1E and 3D). While the underlying mechanisms are an ongoing area of study, these data suggest that increased hypoxia fitness promotes virulence in *A. fumigatus*.

## DISCUSSION

The use of a single strain for the study of pathogenesis and virulence is a common practice in the field of molecular pathogenesis and immunity; however, the increased appreciation for dramatic intraspecies heterogeneity suggests the need to include multiple strains in such studies of virulence. Besides the known clinical implications of pathogen heterogeneity, intraspecies variation provides an outstanding opportunity to better define the virulence mechanisms of a given microbial species. *A. fumigatus* strains, including WT reference strains, clinical isolates, and environmental isolates, have previously been shown to have significant genetic diversity and to display phenotypic heterogeneity *in vitro* and *in vivo* (3, 12–17, 41). Currently, two studies have proposed correlations between *A. fumigatus* isolates, describing a relationship between normoxic fungal growth rate and virulence in the murine neutropenic model of IPA (*n* = 9) (42) and between gliotoxin production and virulence in the *G. mellonella* model of IPA (*n* = 4) (43). However, such studies utilize a relatively low number of exclusively clinical *A. fumigatus* isolates and do not extend the analyses to the diverse group of environmental isolates or virulence in multiple host contexts. Therefore, further analysis of such mechanisms of virulence in a diverse group of isolates may highlight strain-specific virulence factors and host-adaptation strategies, as well as strain-specific host responses, such as those observed between the two “WT” reference strains of *A. fumigatus*, CEA10 and AF293 (44, 45). Heterogeneity in virulence has been observed for several other pathogenic fungi, including *Cryptococcus neoformans* (46), *Paracoccidioides brasiliensis* (47, 48), *C. albicans* (49), and *Histoplasma capsulatum* (50); however, for most fungi, the possible contributing mechanisms have yet to be fully defined. Laboratory passaging of *C. neoformans* serotype A strain H99 results in genomic alterations yielding several phenotypically diverse lineages, several of which are avirulent (46). Similarly, *P. brasiliensis* isolates are also reported to display virulence heterogeneity, and individual isolates lose virulence as a result of *in vitro* laboratory passaging (47), partially as a result of reduced α-1,3-glucan content of the cell wall (48). Moreover, virulence and *in vivo* phenotypic heterogeneities have also been observed in subsets of *C. albicans* strains characterized by white or opaque colonies (49). These observations of phenotypic and virulence heterogeneity in medically important fungi highlight the broad implications of recognizing diversity within a species and underscore the opportunity to define novel mechanisms of virulence in multiple strains of *A. fumigatus.*

Here, we report significant heterogeneity in *A. fumigatus* virulence between two widely used laboratory “WT” strains, AF293 and CEA10, in the triamcinolone murine model of IPA. This significant difference in virulence between the two strains is dependent on the host immune status, as the strains display nearly identical virulence levels in a murine chemotherapeutic model of IPA, where the host is largely leukopenic. This is not the first instance of reported variation between these two strains, as Rizzetto and colleagues (17) reported a number of differences between them in an immunocompetent murine model, which included differences in the inflammatory response and increased pulmonary fungal burden and mortality in CEA10-challenged mice compared to AF293-challenged mice. Analysis of the cytokine profiles induced by each strain revealed that CEA10 elicits a more potent interleukin-17 (IL-17) response by dendritic cells *in vitro* and in the immunocompetent murine model, while AF293 drives a higher production of IL-10, IL-1β, and gamma interferon (IFN-γ), further suggesting that the immune response during an infection is strain dependent (17).

Comparisons of these strains at the genome level reveal that although 98% of each genome can be aligned with high identity (99.3 to 99.8%), 1.4% and 2.3% of AF293 and CEA10, respectively, harbor unique genes. Furthermore, 41 orthologous gene pairs between the two strains do not share 100% identity, and alignment of some of these genes shows identities as low as 37% (51). Taken together, the influence of the variable host environment and the genetic heterogeneity of *A. fumigatus* strains presents multiple opportunities to better understand causative mechanisms underlying virulence in the increasingly common nonneutropenic patient population.

Building upon the known heterogeneity in these “WT” strains, our study demonstrates that AF293 and CEA10 have significantly different fitness levels in low-oxygen (hypoxic) environments when normalized to their fitness in normal-oxygen (normoxic) environments. This ratio of hypoxia fitness correlates with the significant variation in virulence between these two strains. CEA10 not only is more virulent in the triamcinolone murine model of IPA but also displays significantly increased hypoxia fitness as measured by total biomass and colony diameter. This observation led us to hypothesize that increased hypoxia fitness drives increased virulence in host contexts characterized by extensive hypoxia at the site of infection. Hypoxia is a known characteristic of host microenvironments for infections caused by pathogenic organisms, including *Mycobacterium tuberculosis* and *Histoplasma capsulatum* (52–55). Hypoxic lesions have been observed in several tuberculosis animal models, including rats, guinea pigs, rabbits, and nonhuman primates (53, 55), and have been suggested to serve as a trigger for *M. tuberculosis* bacteriostasis, a critical stage of dormancy in *M. tuberculosis* pathology (56). Moreover, *H. capsulatum* causes lesions in the murine liver that contain regions of hypoxic tissue and is able to grow *in vitro* at oxygen tensions below 1% (52). We have previously shown hypoxia to also be a component of the murine pulmonary microenvironment during IPA with both the triamcinolone and chemotherapeutic models of immune suppression. Lesions in both models reach tensions likely well below 1% O_2_; however, the triamcinolone-treated mice present with larger and more severe hypoxic lesions than those in the chemotherapeutic model (26).

In order to thrive in such an oxygen-limiting environment, *A. fumigatus* requires the sterol regulatory element-binding protein (SREBP) transcription factor SrbA. We have previously shown SrbA to be essential for *A. fumigatus* virulence and *in vitro* hypoxic growth (33, 34). The requirement for an SREBP transcription factor in virulence is not unique to *A. fumigatus*, as the SrbA homolog, Sre1, of *C. neoformans* is essential for formation of brain lesions in a murine model of cryptococcosis (27, 57). Further, transcriptional analysis of *H. capsulatum* in hypoxia *in vitro* revealed an upregulated profile of genes with binding sites consistent with conserved SREBP motifs, suggesting an SrbA/Sre1-like hypoxia response such as those characterized in *A. fumigatus and C. neoformans* (52). Thus, we conclude that hypoxia is an important feature of several host-pathogen interactions, and a pathogen’s ability to cope with this environmental stress is essential for virulence.

Given the observed differences between commonly utilized “WT” reference strains, we expanded our analysis to include several environmental and clinical isolates to test the hypothesis that increased hypoxia fitness correlates with increased virulence in the triamcinolone model of IPA. We chose 10 environmental isolates, collected from various geographic and ecological niches, and four clinical isolates (Table 1). Interestingly, across these isolates, greater variation in hypoxia fitness was observed among the 10 environmental isolates than among the four clinical isolates. This reduced variation among the clinical isolates may be the result of the intense oxygen-mediated selective pressures within the host. Notably, the clinical isolates do not cluster based on isolation strategies, i.e., bronchial wash versus lung biopsy. Comparing the virulence levels of these clinical and environmental isolates, it becomes clear that virulence in the triamcinolone model, as measured by median survival, correlates strongly with *A. fumigatus* normoxia-normalized hypoxic fitness (H/N) in liquid culture. Importantly, this correlation applies only to the triamcinolone model of IPA and is not present in the chemotherapeutic model, indicating that hypoxia fitness is a virulence determinant specific to the host immune status.

In support of this correlation between hypoxic fitness and virulence, experimental evolution was performed with AF293 and resulted in a strain, EVOL20, which displays increased hypoxia fitness and, importantly, increased virulence in the triamcinolone model of IPA. Similar *in vivo* and *ex vivo* laboratory passaging experiments have proved insightful in studies of other pathogenic fungi, including *Candida* species and *C. neoformans* (58–60). The ability of the *A. fumigatus* evolved strain (EVOL20) to develop increased hypoxia fitness from *in vitro* exposure not only highlights a role for environmental influence on the heterogeneity of *A. fumigatus* strains but demonstrates the strong selective pressure of altered oxygen tensions for highly virulent strains. It is possible that natural environmental conditions, with various oxygen tensions, can generate genetic and phenotypic alterations that predispose some environmental strains to thrive within the host, accounting for at least a portion of the virulence heterogeneity that we have reported. Given the vast environments from which *A. fumigatus* is isolated (water, air, soil, compost, etc.), the various selective pressures of the environments may partially explain the greater variation in phenotypes that we observe among the environmental isolates compared to the clinical isolates. Work is ongoing to characterize the molecular mechanisms responsible for the increased growth in hypoxia and increased virulence of the hypoxia-evolved strain EVOL20.

**FIG 6:**
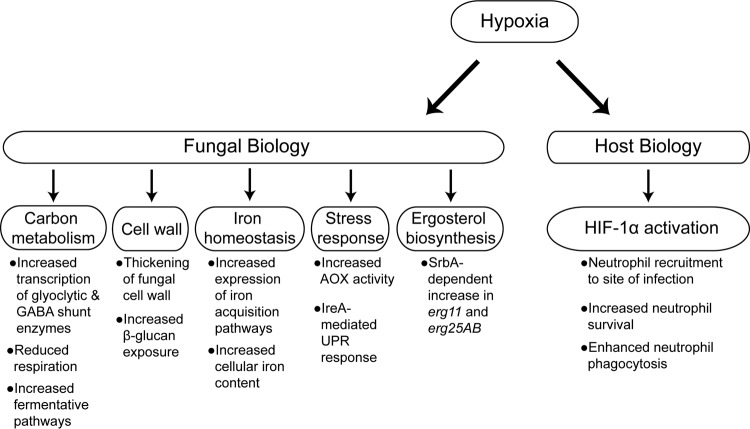
Hypoxia influences a myriad of biological processes in *A. fumigatus* and the host. Hypoxia has been shown to impact several key factors, many of which have identified roles in virulence. Transcriptomics reveals increased expression of glycolytic enzymes as well as enzymes of the γ-aminobutyrate (GABA) shunt (62). Hypoxia has been shown to cause thickening of the cell wall with increased exposure of β-glucan (39). Ergosterol biosynthesis and iron homeostasis are also transcriptionally influenced by hypoxia (64), and increased iron content in the fungi has been demonstrated under hypoxic conditions (63). Last, the cellular response to reactive oxygen species and endoplasmic reticulum stress is altered in hypoxia as shown by increased alternative oxidase activity (AOX) (61) and a hypoxic growth defect in Δ*ireA* (74). UPR, unfolded protein response. Hypoxia-mediated host changes include increased neutrophil recruitment, survival, and effector functions through activation of HIF-1α.

Correlation analyses such as these illustrate a relationship between two phenotypes; however, it does not reveal the causal mechanisms. However, hypoxia acts through several possible mechanisms that may explain the correlation between hypoxia fitness and virulence in the triamcinolone model of IPA. The effects of hypoxia on fungal biology are broad, including changes to virulence-associated mechanisms such as the cell wall (39), cellular metabolism (61–63), iron homeostasis (63, 64), stress mitigation, and responses to host immune cells (39) (Fig. 6). The cell wall directly interacts with the host, and in the triamcinolone model of IPA, where immune cells can drive a hyperinflammatory response to *A. fumigatus* that may result in host damage, this interface is an obvious candidate to drive differences in virulence across strains.

Previous studies have shown that growth in hypoxia results in thickening of the cell wall with increased β-glucan exposure, and cells grown in hypoxia elicit a more potent inflammatory response from immune cells (39). While we did not observe any obvious patterns of differential susceptibility to cell wall-perturbing agents across our isolates under hypoxic conditions, detailed cell wall composition analyses and MAMP exposure are needed to explore this hypothesis further. Hypoxia also affects cellular metabolism of *A. fumigatus*, increasing the transcription and translation of glycolytic enzymes (62, 63) and the use of fermentative pathways (65) while decreasing mitochondrial respiration (61). Evidence in *Saccharomyces cerevisiae* suggests that decreased electron flux through the electron transport chain paradoxically increases the generation of reactive oxygen species (ROS) within the cell (66); thus, mechanisms to deal with redox stresses under hypoxia are required. To this end, the activity of the alternative oxidase is increased in hypoxia, offering a potential mechanism to combat an increase in cellular ROS under these conditions (61). Hypoxia also affects metal ion metabolism, with increased transcription of iron acquisition pathways (64) and increased iron and other critical metal content in cells under low-oxygen conditions (63). These cellular changes, as well as potential unexplored effects of hypoxia, represent possible mechanisms that drive virulence in strains that are well adapted to low-oxygen conditions, and each strain may employ one or several of these mechanisms to thrive within the host environment. Thus, further studies are warranted to understand the causative links between hypoxia and virulence and how these links vary between strains.

In addition to these effects of hypoxia on *A. fumigatus*, low oxygen also affects the host response through modulation of host cell function. Hypoxia has a myriad of known effects on myeloid cell function, including the well-studied effects of the hypoxia-inducible transcription factor HIF-1α. HIF-1α coordinates the cellular response to low-oxygen stress, such as that encountered by neutrophils in the hypoxic microenvironment of infected tissues. In particular, HIF-1α regulates neutrophil recruitment down an oxygen gradient to regions of low-oxygen tensions through the upstream regulation of β_2_-integrin-mediated diapedesis (67). Once at the hypoxic lesion, the critical survival of neutrophils is also HIF-1α coordinated, as demonstrated by reduced survival of HIF-1α-deficient neutrophils following *in vitro* hypoxia exposure (68). Hypoxia has also been shown to have HIF-1α-dependent effects on neutrophil effector function through regulation of phagocytosis and bactericidal activity (69). We have also previously shown that HIF-1α is required for host protection against IPA, due to inefficient neutrophil recruitment at the site of infection (70). Intriguingly, triamcinolone treatment strongly reduces HIF-1α nuclear levels and target gene expression in the lung during *A. fumigatus* challenge (70). Together, the effects of low oxygen on fungal and host-specific processes and the host-pathogen interactions shape the disease progression, severity, and outcome of a given host. Therefore, the specific host environment, in conjunction with fungal genotype and phenotype, must be considered when assessing the host-pathogen interaction.

The correlation between the hypoxic growth and virulence of *A. fumigatus* strains not only highlights the clinically relevant heterogeneity between *A. fumigatus* isolates from the environment and the clinic but also suggests a role for the selective pressures of the environment on the virulence of the organism, where those environments that are oxygen poor may select for strains that are better able to thrive in the hypoxic microenvironment of the infected lung. These data also serve to illustrate that IPA pathology may be directly influenced by the strain of *A. fumigatus* and that the pathology of infection cannot be described merely in terms of the host status and immune response as described previously (71). Future work in *A. fumigatus* virulence studies should consider strain variation in identification of virulence determinants to encompass a clinically relevant cohort of isolates and expand opportunities to develop novel therapeutic approaches in specific patient-fungal interactions.

## MATERIALS AND METHODS

### Strains and media. 

Strains used in this study are listed in Table 1. All strains were stored as conidial suspensions in 50% glycerol at −80°C. Strains were grown on 1% glucose minimal medium (GMM) (72) at 37°C, and conidia were collected in 0.01% Tween for *in vitro* assays. For animal studies, spores were collected in 0.01% Tween and then resuspended in sterile phosphate-buffered saline (PBS). For all solid media, 1.5% agar was added prior to autoclaving.

### Serial passaging of *A. fumigatus* AF293 through a low-oxygen environment. 

The “WT” lab-adapted strain AF293 was grown for 4 days at 37°C in 0.2% O_2_ and 5% CO_2_ on GMM without FeSO4⋅7H_2_O to mimic the oxygen- and nutrient-poor microenvironment of the host. The population of conidia was then subcultured/passaged for 20 generations for a total of 80 days of hypoxic growth. The population of conidia was then single spored, and a single spore was selected based on fitness in hypoxia for further analyses. This strain, EVOL20, was maintained under the passaging conditions prior to further analyses.

### Growth assays. 

Radial growth was quantified by point inoculating 10^3^ conidia on GMM plates at 37°C in normoxia (~21% O_2_, 5% CO_2_) and hypoxia (0.2% O_2_, 5% CO_2_). Colony diameter was measured every 24 h for 4 days and reported as the average from three biological replicates per strain. Fungal biomass was quantified by measuring the dry weight of fungal tissue from 5 × 10^7^ conidia grown in 100 ml liquid GMM with shaking at 200 rpm for 48 h in normoxia (~21% O_2_) and hypoxia (0.2% O_2_, 5% CO_2_). Liquid biomass is reported as the average from three biological replicates per strain. Hypoxic conditions were maintained using an Invivo_2_ 400 hypoxia workstation (Ruskinn Technology Limited, Bridgend, United Kingdom) with a gas regulator and 94.8% N_2_.

### Cell wall-perturbing agents. 

Serial dilutions of conidia were prepared to create dilutions of 10^5^ conidia to 10^2^ conidia/2 µl. Conidial dilutions were plated on GMM containing 1 mg/ml Congo red (Sigma; catalog no. C6277) or 25 µg/ml calcofluor white (fluorescent brightener 28; Sigma; catalog no. F3543). Plates were incubated in normoxia (~21% O_2_, 5% CO_2_) or hypoxia (~0.2% O_2_, 5% CO_2_) for 48 h. Images are representative of biological triplicates.

### Survival analysis.

The virulence of the *A. fumigatus* strains was assessed in two murine models of IPA, the chemotherapeutic model and the triamcinolone model. Mice were housed in autoclaved cages at 4 mice per cage with HEPA-filtered air and autoclaved food and water available *ad libitum*. For the chemotherapeutic murine model, outbred CD-1 female mice (Charles River Laboratories, Raleigh, NC), 6 to 8 weeks old, were immunosuppressed with intraperitoneal (i.p.) injections of cyclophosphamide (Baxter Healthcare Corporation, Deerfield, IL) at 150 mg/kg of body weight 48 h before fungal inoculation and 72 h after fungal inoculation, along with subcutaneous (s.c.) injections of 40 mg/kg Kenalog-10 (triamcinolone acetonide; Bristol-Myer Squibb, Princeton, NJ) 24 h before fungal inoculation and 6 days after fungal inoculation. For the murine triamcinolone model, outbred CD-1 female mice, 6 to 8 weeks old, were treated with 40 mg/kg Kenalog-10 by s.c. injection 24 h prior to fungal inoculation.

For both murine models with the “WT,” environmental, and clinical isolates, conidial suspensions of 2 × 10^6^ conidia were prepared in 40 µl sterile PBS and administered to mice intranasally while under isoflurane anesthesia. Mock mice were given 40 µl PBS without fungal spores. Mice were monitored three times a day for signs of disease for 14 days postinoculation. Virulence of EVOL20 and parental AF293 inocula from passaging conditions was assessed in the triamcinolone murine model, where mice were immunosuppressed as previously described and received a low-dose conidial suspension of 10^5^ conidia in 40 µl sterile PBS 24 h after Kenalog-10 injection. Survival was plotted on Kaplan-Meier curves, and statistical significance between curves was determined using the Mantel-Cox log rank.

### Histopathology.

Outbred CD-1 mice, 6 to 8 weeks old, were immunosuppressed and intranasally inoculated with conidia as described above for the chemotherapeutic and corticosteroid murine models. Three mice in each group were inoculated with either 2 × 10^6^ AF293 conidia, 2 × 10^6^ CEA10 conidia, 1 × 10^5^ EVOL20 conidia, or 1 × 10^5^ AF293 conidia from the passaging conditions as described above. Mice were sacrificed 72 h postinoculation. Lungs were perfused with 10% buffered formalin phosphate before removal and then stored in 10% buffered formalin phosphate until embedding. Paraffin-embedded sections were stained with hematoxylin and eosin (H&E) and Gomori methenamine silver (GMS). Slides were analyzed microscopically with a Zeiss Axioplan 2 imaging microscope (Carl Zeiss Microimaging, Inc., Thornwood, NY) fitted with a QImaging Retiga-SRV Fast 1394 RGB camera. Analysis was performed with Phylum Live 4 imaging software.

### Ethics statement.

We carried out our animal studies in strict accordance with the recommendations in the *Guide for the Care and Use of Laboratory Animals* (73). The animal experimental protocol was approved by the Institutional Animal Care and Use Committee (IACUC) at Dartmouth, College (federal-wide assurance number A3259-01).

### Statistics and correlation analysis.

All statistical analyses were done with Prism 5 software (GraphPad Software Inc., San Diego, CA). Correlations were calculated using the Spearman rank order correlation analysis for nonparametric data. All error bars represent standard errors of the means.

## SUPPLEMENTAL MATERIAL

Figure S1  Analysis of isolates on cell wall-perturbing agents in normoxia and hypoxia. Serial dilutions of each strain (WT, clinical, and environmental isolates) on GMM with 1 mg/ml Congo red (CR) or 25 µg/ml calcofluor white (CFW) in normoxia (A) or hypoxia (B). Download Figure S1, TIF file, 15.8 MB
