# Supplementary figures and images for: Heterogeneity among Isolates Reveals that Fitness in Low Oxygen Correlates with Aspergillus fumigatus Virulence
Source: mBio. 2016 Sep 20;7(5):e01515-16. doi: 10.1128/mBio.01515-16 (PMC5040115; doi:10.1128/mBio.01515-16)

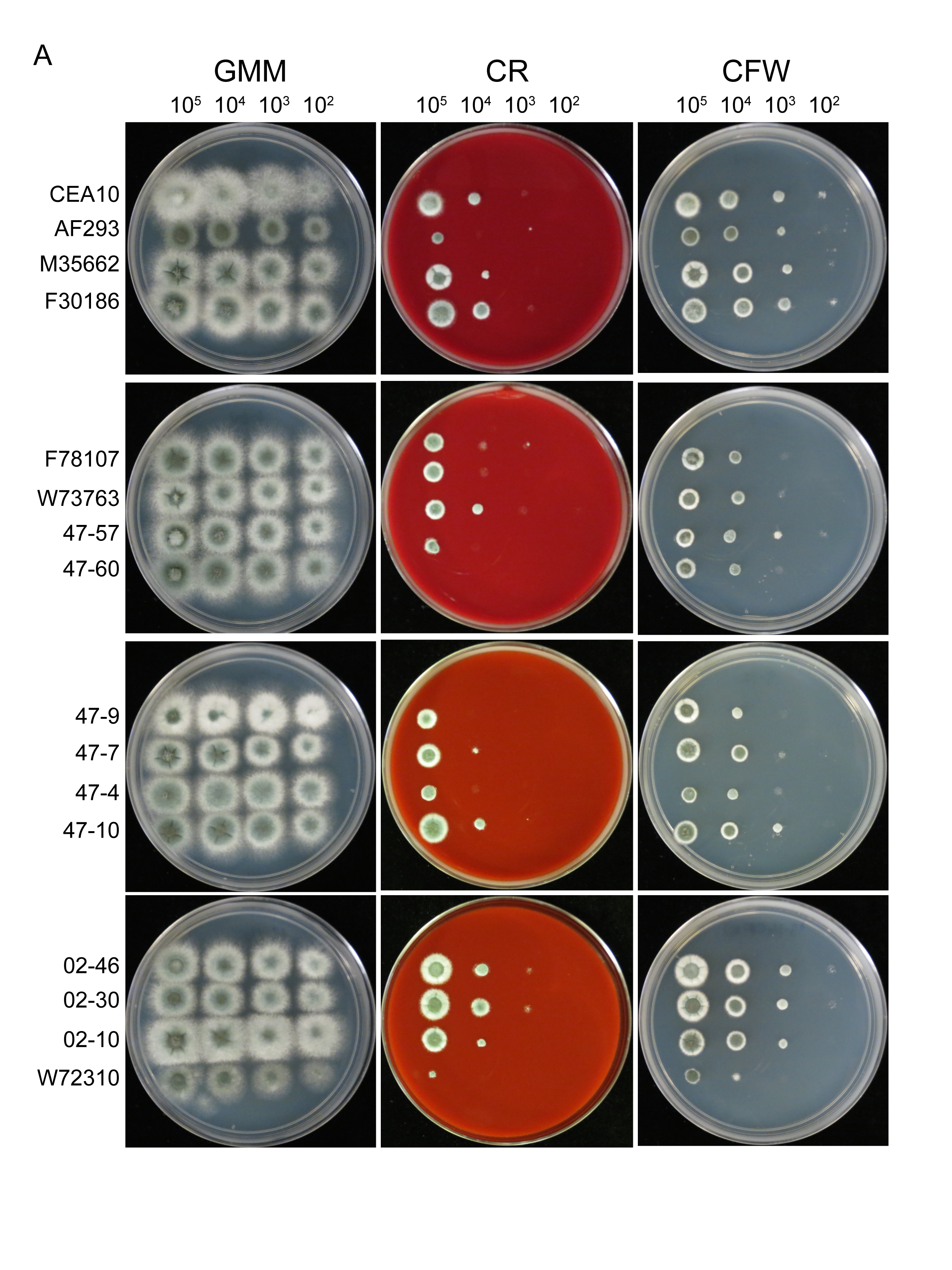

Supplement: Figure S1 — Analysis of isolates on cell wall-perturbing agents in normoxia and hypoxia. Serial dilutions of each strain (WT, clinical, and environmental isolates) on GMM with 1 mg/ml Congo red (CR) or 25 µg/ml calcofluor white (CFW) in normoxia (A) or hypoxia (B). Download [file mbo004162991sf1.tif]
